# Editorial: Advances in tick-borne pathogens and their interactions with hosts

**DOI:** 10.3389/fvets.2024.1505133

**Published:** 2024-10-17

**Authors:** Yali Sun, Zedong Wang, Charles Byaruhanga, Yanan Wang, Mingming Liu

**Affiliations:** ^1^College of Agriculture and Animal Husbandry, Qinghai Provincial Key Laboratory of Pathogen Diagnosis for Animal Diseases and Green Technical Research for Prevention and Control, State Key Laboratory of Plateau Ecology and Agriculture, Qinghai University, Xining, China; ^2^Department of Infectious Diseases, Center of Infectious Diseases and Pathogen Biology, Key Laboratory of Organ Regeneration and Transplantation of the Ministry of Education, The First Hospital of Jilin University, Changchun, China; ^3^Department of Veterinary Tropical Diseases, Faculty of Veterinary Science, University of Pretoria, Onderstepoort, South Africa; ^4^Shanghai Veterinary Research Institute, Chinese Academy of Agricultural Sciences, Shanghai, China; ^5^School of Basic Medicine, Hubei University of Arts and Science, Xiangyang, China

**Keywords:** tick-borne pathogens, *Babesia*, *Theileria*, *Dermacentor nuttalli*, *Ixodes gibbosus*

Ticks serve as vectors for a wide array of infectious microorganisms which are agents for tick-borne diseases that significantly impact both human and animal health. This Research Topic aims to deepen our understanding of pathogen transmission and interactions between arthropod vectors and their vertebrate hosts. Four articles were accepted for publication in the Research Topic “*Advances in tick-borne pathogens and their interactions with hosts*.”

Yin et al. systematically analyzed the genetic diversity of *Babesia gibsoni*, revealing critical insights into its population structure. Their study found low genetic differentiation and high gene flow within populations in a single continent, while greater differentiation was observed across continents. Neutrality tests suggested that these populations have undergone demographic expansion. This enhances our understanding of the genetic diversity and complex dynamics of *B. gibsoni*, which is crucial for developing effective control strategies against canine babesiosis.

Altay et al. identified A and E genotypes of *Theileria equi* in grazing horses from Kyrgyzstan, shedding light on the epidemiology of these genotypes. They also detected *Anaplasma phagocytophilum, A. capra*, and hemotropic mycoplasmas in the horse population, with *A. capra* being a relatively novel species and believed to have a global distribution. Further large-scale studies are needed to fully understand the prevalence, distribution, and pathogenicity of this pathogen in horses.

Ma et al. characterized the life cycle of *Dermacentor nuttalli* collected from the Qinghai-Tibetan Plateau under laboratory conditions for the first time. They identified two species of SFG *Rickettsia* in the midgut and salivary glands of both male and female ticks from the field and the first laboratory generation. The study highlights discrepancies between laboratory and natural conditions that may affect tick survival and lifecycle, along with the importance of considering seasonal diapause in relation to pathogen colonization. This research provides new insights into host-tick-pathogen interactions in the Qinghai-Tibetan Plateau ecosystem.

Diakou et al. documented clinical and epidemiological findings of tick paralysis in domestic animals in Cyprus, particularly in goats, sheep, dogs, and cats. Affected animals, free from other neurological diseases and exhibiting normal blood parameters, recovered quickly after tick removal primarily from the head and neck. Cases predominantly occur in the Akamas peninsula from September to March, following a 3- and 7-year periodic cycle of varying severity and animal loss. These cycles may be influenced by external factors, self-oscillations, or a combination of both. Recent reports identified a tick species as *Ixodes gibbosus*. The study highlights the urgent need to characterize the specific toxins responsible for tick paralysis and to develop a vaccine that could significantly reduce losses in small ruminants, particularly in free-ranging farming systems common in Cyprus and other areas.

In summary, this Research Topic has provided valuable insights into recent advancements in the study of tick-borne pathogens and their interactions with hosts. Each aspect presents opportunities for further investigation, essential for enhancing health outcomes for both humans and animals.

